# Securing Cryptographic Chips against Scan-Based Attacks in Wireless Sensor Network Applications

**DOI:** 10.3390/s19204598

**Published:** 2019-10-22

**Authors:** WeiZheng Wang, Zhuo Deng, Jin Wang, Arun Kumar Sangaiah, Shuo Cai, Zafer Almakhadmeh, Amr Tolba

**Affiliations:** 1 College of Computer and Communication Engineering, Changsha University of Science and Technology, Changsha 410114, China; peakexpe@csust.edu.cn (W.W.); dz5019@stu.csust.edu.cn (Z.D.); 005861@csust.edu.cn (S.C.); 2 Hunan Provincial Key Laboratory of Intelligent Processing of Big Data on Transportation, Changsha University of Science and Technology, Changsha 410114, China; 3 School of Information Science and Engineering, Fujian University of Technology, Fuzhou 350118, China; 4 School of Computing Science and Engineering, Vellore Institute of Technology (VIT), Vellore 632014, Tamil Nadu, India; sarunkumar@vit.ac.in; 5 Computer Science Department, Community College, King Saud University, Riyadh 11437, Saudi Arabia; zalmakhadmee@ksu.edu.sa (Z.A.); atolba@ksu.edu.sa (A.T.); 6 Mathematics and Computer Science Department, Faculty of Science, Menoufia University, Shebin-El-kom 32511, Egypt

**Keywords:** cryptography, wireless sensor networks, hardware security, scan-based attack

## Abstract

Wireless sensor networks (WSN) have deeply influenced the working and living styles of human beings. Information security and privacy for WSN is particularly crucial. Cryptographic algorithms are extensively exploited in WSN applications to ensure the security. They are usually implemented in specific chips to achieve high data throughout with less computational resources. Cryptographic hardware should be rigidly tested to guarantee the correctness of encryption operation. Scan design improves significantly the test quality of chips and thus is widely used in semiconductor industry. Nevertheless, scan design provides a backdoor for attackers to deduce the cipher key of a cryptographic core. To protect the security of the cryptographic system we first present a secure scan architecture, in which an automatic test control circuitry is inserted to isolate the cipher key in test mode and clear the sensitive information at mode switching. Then, the weaknesses of this architecture are analyzed and an enhanced scheme using concept of test authorization is proposed. If the correct authorization key is applied within the specific time, the normal test can be performed. Otherwise, only secure scan test can be performed. The enhanced scan scheme ensures the security of cryptographic chips while remaining the advantages of scan design.

## 1. Introduction

In recent years, wireless sensor networks (WSNs) have been widely used in smart communities because of their potential advantages. They can supply the distributed communication platform for various applications, such as intelligent transportation, smart home, industrial monitoring, logistics, health care and so on [1,2]. Particularly, through the integration with Internet of Things (IoT), WSNs are playing a more important role and will benefit mankind more significantly [3,4,5,6,7,8,9]. However, the rapid deployment of WSNs through various networks results in different security and privacy concerns and challenges [10,11,12]. Hence, security and privacy protection for WSNs becomes particularly important.

In such a situation, cryptography is widely exploited to ensure the integrity and security of data and information in WSN. Cryptographic algorithms are often applied in specific circuits to fulfill the demand of high throughput in resource-constrained environments [13]. Unfortunately, integrated circuits also face various security threats [14,15,16]. The scan-based side-channel attack is the most common type of security threats. To guarantee the correctness of the data encrypted/decrypted, the faulty crypto chips must not be used in a cryptography system. Hence, rigorous testing for crypto chips is essential. Whereas, the increase in design complexity brings great challenge to integrated circuit (IC) testing. In this background, design for testing (DFT) methodology has been proposed and scan design is the most prevalent DFT technique, which replaces the original D flip-flops in the circuit with scannable cells and connects them into one or multiple scan chains. Scanning-in/-out operation of the scan chains endows the chip with full controllability and observability and thus scan design significantly decreases the complexity of automatic test pattern generation (ATPG) and cuts down the test cost. However, scan design also provides a side channel for attackers to steal the sensitive information of cryptographic chips. Nowadays, scan-based attacks thread the security of cryptographic chips seriously. In the past decades, scan-based attacks have been deeply studied by the researchers. The scan be divided into two general categories as follows:

(1) Mode-switching attacks

Advanced Encryption Standard (AES) has become the most common cryptographic algorithm due to its high security. Hitherto, no brute-force attack targeting it has been reported. Nevertheless, the scan-based attacks conducted on the AES chip have been proposed to obtain the sensitive information such as the cipher key [17]. The scan design gives attackers a side-channel to crack a crypto chip. In a crypto chip, the encryption result generated after each round is stored in a state register, which is included in scan chains. For AES with 128-bit key, the encryption result generated after the tenth round has strong enough resistance to any mathematical attacks, but the intermediate result obtained after the first round can be analyzed to deduce the secret key [18]. The attackers first apply the crafted plaintext and execute AES for only one round of encryption. Then, they switch the AES chip to test mode, and observe the intermediate round result by scanning out the values of scan chains. This process is repeated by using pairs of plaintexts whose Hamming distance is 1. Once the Hamming distance of the intermediate results of two paired plaintexts satisfies specified conditions, one key byte can be determined by the adversaries. On average, 256 pairs of plaintexts are required to crack a cipher key with the length of 128 bits. 

Aside from the scan attack on AES chips, a scan-based differential attack on Elliptic Curve Cryptography (ECC) chip is proposed in [19]. The key operation of ECC algorithm is the point multiplication, which is executed iteratively using a different part of the cipher key at a time. With the aid of scan chains, the adversary can obtain the results of intermediate multiplications and retrieve the cipher key. Besides, the researchers have reported that scan-based noninvasive attacks can also be performed on other cryptographic chips, e.g., Rivest-Shamir-Adleman (RSA) chips [20] and Data Encryption Standard (DES) chips [21]. 

In addition, the authors of [22,23] enhanced the scan attacks and applied them to advanced DFT architectures. In industry some advanced solutions, e.g., test stimulus decompressor [24] and test response compactor [25], are usually deployed to reduce test data volumes. In the past, they were considered as a natural protection mechanism of crypto chips, but the researches in these papers prove that the differential scan attack can still be conducted on crypto-processors even with the insertion of advanced DFT architectures.

(2) Test-mode-only attacks

The scan attacks above require the switching between the functional mode and test mode. Hence, they can be easily overcome by countermeasures based on resetting the circuit at the time of mode switching. Ali S.S. et al. [26] propose a novel scan-based attack on AES which can be carried out only in test mode. In this scan-based attack, the AES plaintexts are inputted through the boundary scan chain. The encryption result of the first round is captured into scan chains at the capture phase of test mode and then observed at scan output by the shifting operation of scan chains. The attack model is developed to thwart the secure techniques based on mode switching reset. The authors also extend their attack model to decompressor-based scan architectures [27].

In this paper, we aim to propose a secure and low-overhead scan methodology for resisting scan-based attacks. The main motivation of our research work can be described as follows:

- The proposed secure scan methodology will achieve complete protection against all categories of scan-based attacks. This can fully ensure the security for cryptographic chips in WSN applications.
- The advantages of scan design can be retained while improving the security of chips. In the proposed technique, only secure scan tests can be performed by unauthorized users, i.e., the cipher key is protected in test mode and the secret information is cleared when the circuit is switched from the normal mode to test mode. Just like standard scan design, the proposed scheme will provide full testability for the circuit under test (CUT) and make online testing executable for the authorized users.
- Under the prerequisite of security and testability guaranteed, a very lightweight hardware mechanism is proposed to extend the application range of the proposed scheme, especially for resource-constrained environments such as WSN. Based on this consideration, the proposed scheme designs a smart automatic test control unit and a small test authorization circuitry.

The rest of this manuscript is organized as follows: Section 1 introduces some preliminaries including the standard scan design and the existing countermeasures thwarting scan attacks. In Section 2, the proposed scheme for securing crypto chips against scan attacks is discussed in details. An improved secure strategy is presented in Section 3. Section 4 presents performance analysis on the proposed enhanced scheme. Finally, Section 5 concludes this manuscript.

## 2. Scan Design and Countermeasures Thwarting Scan Attacks

### 2.1. Scan Design

**Figure 1:**
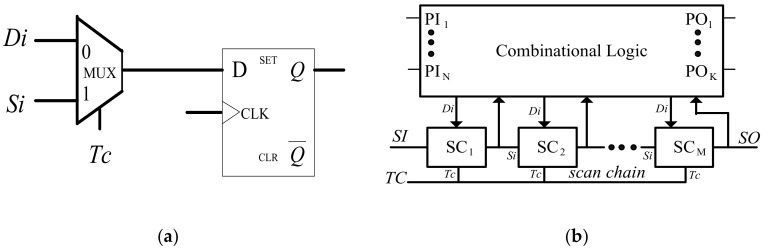
Scan design: (**a**) A standard scan cell (SC); (**b**) A full-scan circuit with single scan chain.

Initially, scan design was presented for improving the testability of sequential circuits. It modifies the D flip-flop into a scan cell by inserting a multiplexer to its input port. As shown in Figure 1a, with the control of a test control (*Tc*) signal, the 2-to-1 multiplexer selects either the data input (*Di*) or the scan input (*Si*). *Di* is the original input of D flip-flop.

As shown in Figure 1b, a scan chain is constituted by successively connecting the output of a scan cell to the *Si* of another scan cell. The *Si* of the head-most scan cell is tied to a scan input port (*SI* port) while the *Q* output of the last scan cell is tied to a scan output port (*SO* port). Arbitrary values can be loaded serially into the scan chain through *SI* port and the state of the scan chain can be shifted out through *SO* port. Hence, the initial state of the circuit is able to be set as required and the response is also able to be observed by using of scan chains, and the high testability is achieved. In order to decrease test time, multiple scan chains are often utilized to achieve the parallel loading of test data.

The system test control signal (labeled as *TC*) drives the *Tc* input of all the scan cells. When *TC* is set to ‘0’, the circuit is running in the normal mode. If *TC* = 1, then the circuit enters into the test mode. During this period, the test pattern is scanned bit-by-bit into scan chains while the values of scan chains is scanned out. As long as the test pattern is completely delivered into the scan chains, *TC* is set to ‘0’ for 1 (used in stuck-at fault testing) or 2 (used in launch-on-capture delay testing) clock cycles. The current response of the circuit is captured into the scan cells via the data input *Di* at a valid clock edge. The clock cycle(s) is (are) also referred as “capture mode”. As *TC* goes to ‘1’, the test response stored in the scan chains is scanned out via *SO* port while the next test pattern is loaded into the scan chains via *SI* port. The procedure is repeated until the CUT is fully tested.

### 2.2. Countermeasures Thwarting Scan Attacks

Because standard scan design brings serious threat to the security of cryptographic chips, researchers have presented various secure DFT solutions in recent years. Initially, resetting the chip when it is switched from normal mode to test mode was exploited to safeguard against scan attacks [28]. However, the attacker is able to conduct a test-mode-only attack on the protected chip as described in [26]. The countermeasure in [17] divides the working mode of the circuit into the secure and insecure mode. If the circuit enters the secure mode after a system reset, the encryption operation can be normally performed. If the circuit first enters the insecure mode after a system reset, the circuit testing can be launched but the cipher key is kept apart from the cryptographic module to avert being cracked. The design can jump to the secure mode from the insecure mode, however, the opposite jump is prohibited. In [29], the authors disable completely the switch between the test mode and normal mode and protect the cipher key in test mode. This can be reshuffled only after the system reset. Another countermeasure based on protection of cipher key is proposed in [30] for boundary scan design. These countermeasures in [28,29,30] are resistant to test-mode-only attack, but they also cause that the faults on the round key generation unit cannot be detected and thus reduce the reliability of cryptographic chips that pass the testing.

Secure techniques based on restricted access to scan chains are proposed in [31,32,33]. The scheme proposed in [31] inhibits the normal scan operation if the user is unauthorized. The technique proposed in [32] manages access to scan architecture by specifying and verifying multilevel access permission and restriction to instruments associated with the reconfigurable scan networks. Novak F. et al. [33] modify the TAP (Test access port) controller to restrict access to scan chains. The modified TAP controller has two states: locked and unlocked state. Only the user with correct password can unlock the TAP controller and be granted the full access to scan chains. 

Partial Scan Design is a quite attractive approach for protecting crypto chips since the state registers involving the sensitive information are excluded from the scan chains and the intermediate encryption results are no longer accessible from scan output [34,35]. However, this DFT methodology compromises the controllability and observability of circuits and may result in some loss in fault coverage. For this reason, the scheme proposed by Chen et al. provides the balance of security and testability using configurable scan architecture [36]. 

The countermeasures based on data obfuscation modify the scan-in and/or scan-out data with an unpredictable way [37,38,39,40,41]. Obfuscating the scan data can prevent or mislead the attackers to obtain the correct cipher key. Atobe et al. [37] dynamically configure the connection of the sub-chains to disturb the values observed at the scan output. Nevertheless, the authors of [42] have proven that the sophisticated attackers are still able to perform the scan attack without knowing the order of scan cells. In [38], an extra shift register, the states of which are exploited to control the work mode of some selected scan cells, is inserted into CUT. If the user don’t load the correct key into the shift register, the scan cells controlled by the incorrect bit of the key cannot enter the test mode during testing. As the shifting operation of test data is disturbed, the test patterns fed to scan chains and test responses observed at SO port are obfuscated. Furthermore, the wrong key can make the added shift register cyclically shifted during testing, so the scan cells remaining in the normal mode will dynamically change. The dynamic obfuscation of scan data is achieved by this way. In order to further improve security, Cui A. et al. expand this technique by using the Physical Unclonable Function (PUF) [43] as the key in [40]. Wang et al. [41] insert some XOR gates between scan cells, which are controlled by a Linear Feedback Shift Register (LFSR). Only the designers who know the state sequence of LFSR and the position of XOR gates can apply the desired test patterns and restore the real test responses from the observed values. However, these countermeasures usually incur comparatively large hardware overhead.

Secure designs based on data encryption were developed in [44,45,46,47]. The technique presented in [44] encrypts the test patterns, which are delivered to the IEEE 1500-compliant intellectual property cores, with the Trivium stream cipher. Using a given seed, the stream cipher can generate a pseudo-random keystream that will be XORed with the data to encrypt. The solution in [45] uses the block cipher to encrypt the test data in scan chains. The block cipher encrypts a block of n-bit data at a time with a fixed key stored in the device. If the user does not have the knowledge of block ciphers, he can neither load desired test stimulus into the scan chains nor obtain original test responses from SO port. The block cipher is implemented in different manners in [46] and [47]. Lightweight block ciphers are preferred to achieve a perfect trade-off between security and area penalty. The main drawback of these schemes is that they incur relatively large test time overhead to encrypt/decrypt the test data.

The techniques proposed in [48,49] carry out the online detect of scan attacks by monitoring the user behavior in real time. Once the user behavior is regarded as illegal, the circuit automatically enters a protection mode. The detection method in [48] uses sequence filters arranged on the TAP controller to manage the access to the test infrastructure. It prevents the illegal user from accessing the protected instruments. The authors of [49] proposed a detection scheme using representative sequences of instructions, which represent the illegal operations and are determined at design stage. If the user behavior involves the representative sequences, it is considered as an attack. These techniques are very secure and intelligent. However, they incur very large area penalty, which would limit their application. 

To overcome the limitations of the countermeasures described above, further research is still needed and more practical secure solutions should be developed.

## 3. Secure Scan Scheme Based on Automatic Test Control Unit

**Figure 2:**
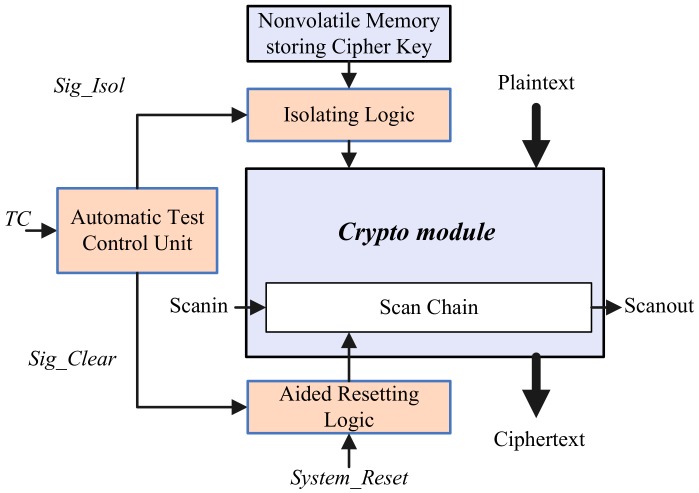
Hardware framework of proposed secure scan test scheme.

In this section, we propose a secure scan design scheme based on automatic test control unit (abbreviated to SSATCU design scheme below). The hardware framework is described in Figure 2. The automatic test control unit has one input, i.e., the system test control signal *TC*, and two output signals *Sig_Isol* and *Sig_Clear.* By setting *Sig_Isol* to the valid value (i.e., ‘1’), the automatic test control unit can isolate the cipher key from the encryption module during scan testing. Besides, when the circuit turns to the test mode, the automatic test control unit also clears the sensitive state stored in scan chains by asserting *Sig_Clear* high. 

At power-on, the system including the automatic test control unit is reset. Afterward, if *TC* = 0, the chip runs in the normal mode. In this mode, the automatic test control unit makes both *Sig_Isol* and *Sig_Clear* invalid. The cipher key can be loaded into the crypto module and the encryption operation can be executed normally. When *TC* goes to ‘1’, the chip is switched to test mode from normal mode. At this time, the automatic test control unit outputs ‘1’ for the signal *Sig_Clear* for one clock cycle. The intermediate encryption results stored in the round register that is part of the scan chain, is cleared by using the aided resetting logic. Since the chip enters into the test mode, *Sig_Isol* remains ‘1’ and the encryption key is isolated by using the additional isolating logic. The additional automatic test control unit guarantees the security of a scan test without exposing the information involving the secret key. The detailed description of the automatic test control unit, key isolating logic, and aided resetting logic is given as follows:

(1) Automatic test control unit

**Figure 3:**
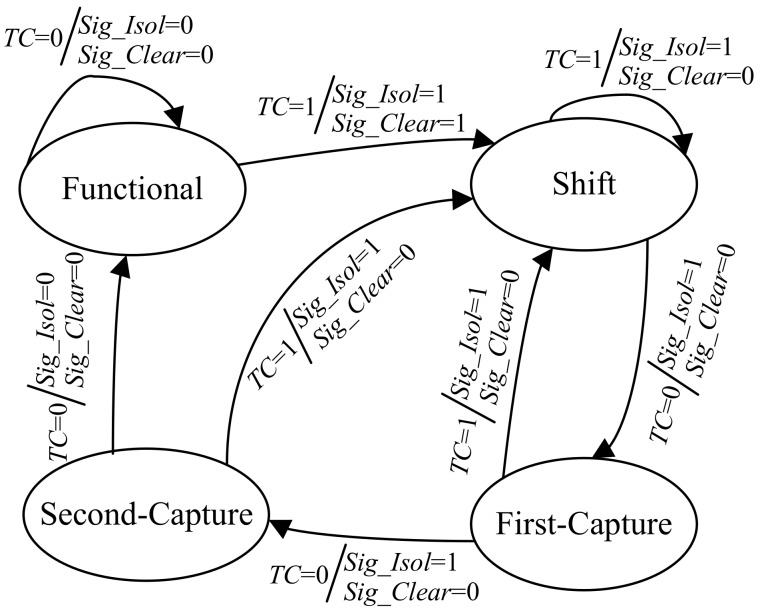
State diagram of automatic test control unit.

The working (clock) cycles of the CUT can be divided into four types: the functional cycles, the shift cycles, the first capture cycle and the second capture cycle. It should be noted that, for the testing that only needs one capture cycle, there are only three types of working cycles (i.e., the second capture cycle is excluded). In order to make the scheme applicable to all kinds of testing including launch-on-capture delay testing, we consider the second capture cycle. The behavior of the automatic test control unit is different in each type of working cycle. Therefore, the automatic test control unit also has four working states, which correspond to the four types of working cycles respectively. The state diagram of the automatic test control unit is shown in Figure 3. The four working states are named as “functional”, “shift”, “first-capture” and “second-capture”. The arrow indicates the direction of the state transition under the specified input condition (i.e., *TC* = 0 or 1) which is given before the symbol ‘/’. The values of the output signals *Sig_Isol* and *Sig_Clear* are given after the symbol ‘/’, which are determined by the current state and *TC*.

When the automatic test control unit is in “functional” state and *TC* is ‘0’, *Sig_Isol* = 0, *Sig_Clear* = 0 and the next state of the automatic test control unit is still “functional” state. When the automatic test control unit is in “functional” state and *TC* is ‘1’, *Sig_Isol* = 1 and *Sig_Clear* = 1. The content of scan chains is flushed away immediately and the cipher key will be isolated in the following test mode. Under this situation, the next state of the automatic test control unit is “shift” state. If *TC* remains ‘1’ in “shift” state, *Sig_Isol* remains ‘1’, *Sig_Clear* returns to ‘0’, and the next state is still “shift” state. If *TC* goes to ‘0’ later, the capture operation is first considered. Hence, *Sig_Isol* and *Sig_Clear* remains ‘1’ and ‘0’, respectively. The next state is “first-capture” state. If *TC* remains ‘0’ in the following clock cycle, the automatic test control unit enters into “second-capture” state. If *TC* returns to ‘1’ in “first-capture” state or “second-capture” state, the automatic test control unit returns to “shift” state and the cipher key keep isolated. When *TC* is ‘0’ for more than three cycles, it is considered that the CUT enters into the normal mode. At this time, *Sig_Isol* becomes ‘0’ and thus the mask of the cipher key is removed. The automatic test control unit returns to the initial “functional” state.

**Figure 4:**
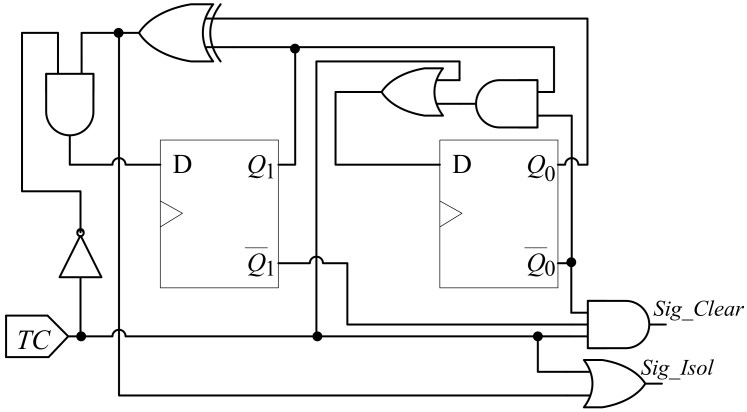
Architecture of the automatic test control unit.

**Figure 5:**
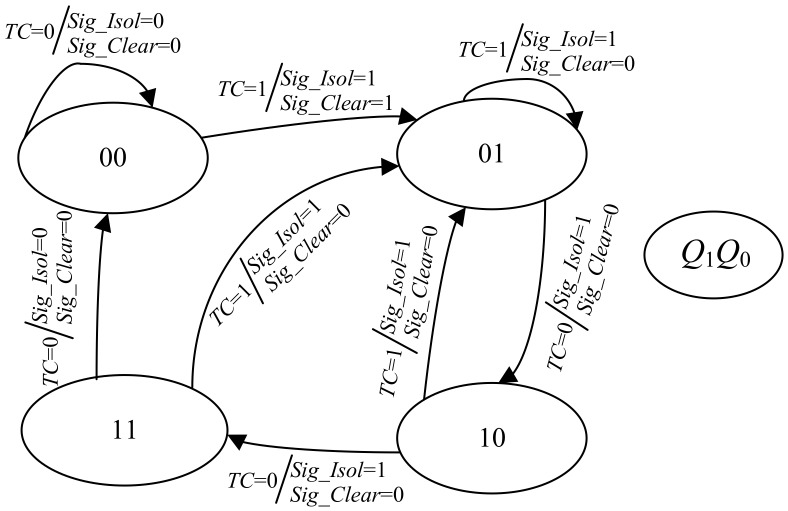
The actual state diagram.

By using the theories of digit circuit design, the automatic test control unit is designed as shown in Figure 4 according to the state diagram in Figure 3. The hardware implementation of automatic test control unit is low-cost, which is only comprised of two D flip-flops and a few logic gates. The actual state diagram corresponding to the hardware implementation of the automatic test control unit is shown in Figure 5. As shown in the figure, the state diagram is as same as the one described in Figure 3. States “00”, “01”, “10” and “11” correspond to the previous “Functional”, “Shift”, “First-capture” and “Second-capture”, respectively.

(2) Aided resetting logic

**Figure 6:**
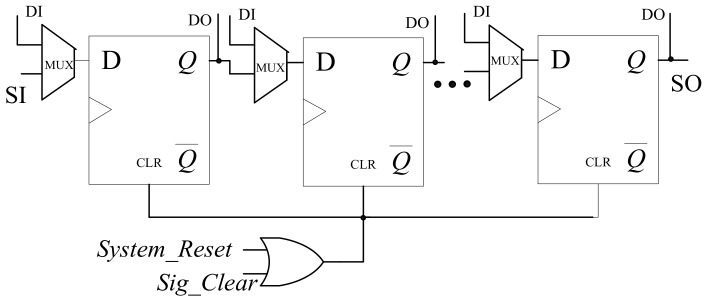
Aided resetting logic.

As illustrated in Figure 6, the aided resetting logic includes only an OR gate. The reset port of each scan flip-flop is controlled by the system reset signal *System_reset* ORed with *Sig_Clear*. When performing the system reset (i.e., *System_reset* = 1), the scan chain can be reset immediately regardless of the value of *Sig_Clear*. At same time, the content of the scan chain can also be cleared by reset operation as long as *Sig_Clear* is ‘1’.

(3) Isolating logic

**Figure 7:**
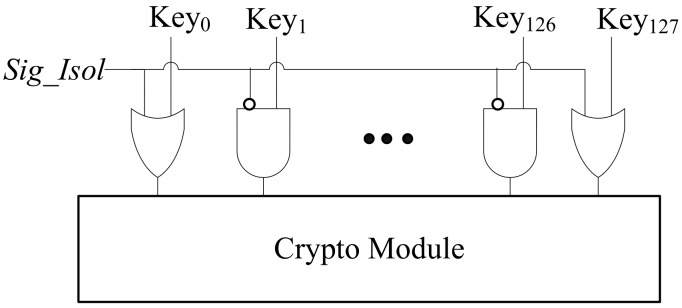
Isolating logic.

The logic circuitry to isolate the key is illustrated in Figure 7. As can be seen from the figure, the secret key bits can be shielded by two ways: using an AND gate and using an OR gate. Part of bits of the secret key are transmitted via an OR gate and then loaded into the crypto module. The other input of the two-input OR gate is driven by *Sig_Isol* that is produced by the automatic test control unit. The rest of bits of the secret key are transmitted via an AND gate whose other input is fed by logical NOT of *Sig_Isol*. These two types of isolating logic can be selected randomly. In normal mode, *Sig_Isol* will be ‘0’ and the extra logic gates are transparent, so every bit of the secret key (i.e., Key_0_, Key_1_, …, Key_126_, Key_127_) can be propagated to the crypto module. Otherwise, if the chip is running in test mode, *Sig_Isol* will be ‘1’and every bit of the secret key will be prevented from passing to the encryption module. Under this situation, the additional AND gates and OR gates output logic ‘0‘ and ‘1’ for the crypto module, respectively. The actual secret key is replaced by the dummy key that will be delivered to the crypto core as encryption key. The chip designer can arbitrarily configure the dummy key by selecting different combination of isolating way for each secret key bit.

The proposed SSATCU design scheme performs the secure scan test, resulting in high security. Even if the attacker performs the encryption operation for one round or one clock cycle in the normal mode and makes the encrypted results stored in the scan chain, he cannot observe the intermediate results at scan output port. This is because that, the SSATCU design scheme clears the state of scan chains when the mode switching occurs. Hence, it can thwart scan attacks based on mode switching. The proposed scheme also isolates the secret key during the whole test process, so test-mode-only scan attacks can be overcome as well. 

However, the SSATCU scheme compromises the testablity of CUT. Firstly, it inhibits the online testing since the state obtained in function operation is protected. Performing online testing can contribute to decrease the test time and meanwhile allow faults that won’t obstruct the functional operation to avoid being detected. Hence, online testing is very important and widely used in semiconductor industry [50]. Secondly, because the dummy key, instead of the real secret key, is propagated to the key generation unit in test mode, some faults in the key generation unit that will obstruct the functional operation cannot be detected. It is not inadvisable to sacrifice the testablity for security. To give consideration to both testablity and security, we propose an improved secure scan design scheme based on automatic test control unit in the next section.

## 4. Improved Secure Scan Scheme Based on Automatic Test Control Unit

In the improved secure scan design scheme based on automatic test control unit (abbreviated to ISSATCU design scheme below), we unlock the test protection described in Section 2 for the authorized users, and only perform the secure scan test for the unauthorized users. 

**Figure 8:**
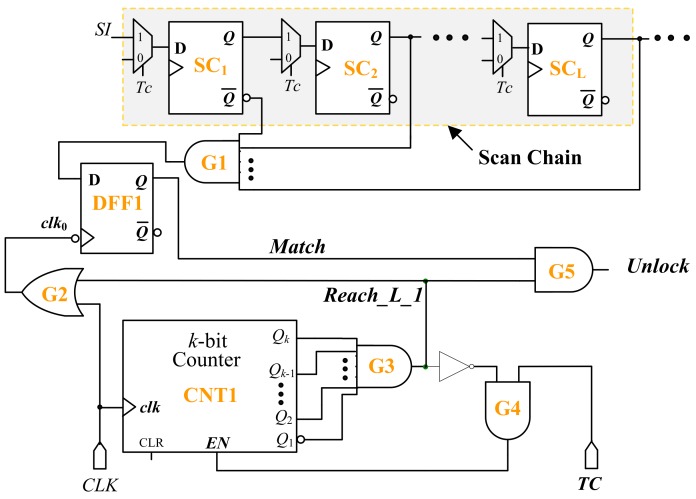
Test authorization mechanism.

**Figure 9:**
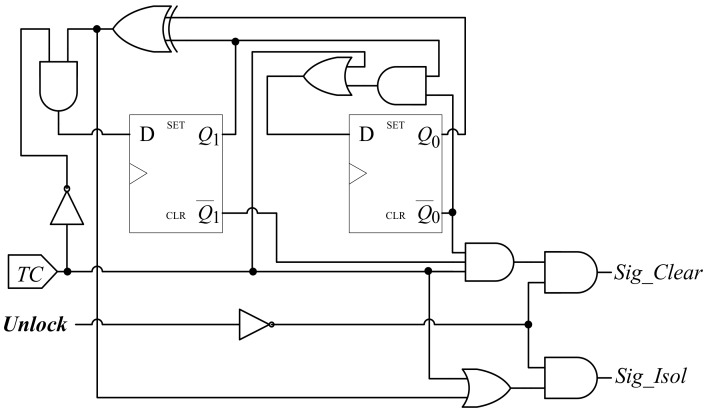
Architecture of improved automatic test control unit.

The test authorization mechanism is shown in Figure 8. In order to reduce the hardware overhead, instead of using an extra shift register, part of the scan chain is selected to store the test authorization key. Assume that, *L* scan cells near scan input pin are selected. At the beginning of testing, the test authorization key should be loaded into the selected part of the scan chain through *SI* port. The test authorization key is used to generate an unlock signal to disable the automatic test control unit, as shown in Figure 9. Either the complementary output $\bar{Q}$ or the output Q of each selected scan cell is fed to a multiple-input AND gate labeled as G1 in Figure 8. If a test key bit is ‘1’, then the output *Q* of the corresponding scan cell is connected to G1. Otherwise, the complementary output $\bar{Q}$ is connected to G1. When loading the correct test authorization key into the scan chain, the output of G1 is ‘1’. The output of G1 will be ‘0’ if at least one bit of the test authorization key is mismatched. The value of G1 will be latched into a D flip-flop labeled as DFF1 when the test authorization key is completely delivered. It should be noted that, unlike other flip-flops, DFF1 is a falling-edge triggered D flip-flop. A *k*-bit (*k* = ⌊log_2_(*L +* 1)⌋ + 1) counter CNT1 is employed to record the clock cycles during loading the test key. CNT1 starts counting from all zeros when the CUT enters into the test mode (i.e., *TC* = 1). After *L* clock cycles of *CLK*, the test authorization key is completely loaded. At the following falling edge of CLK, the output of G1 determined by the authorization key will be stored into DFF1. At (*L* + 1)^st^ clock cycle, the state of CNT1 becomes *L +* 1 (i.e., the binary sequence *Q*_k_*Q*_k-1_…*Q*_2_*Q*_1_ denotes the decimal number *L* + 1). The inputs of the AND gate G3 should be elaborately designed to make that the output of G3 is ‘1’ when the state of CNT1 is *L +* 1. For example, let’s assume that *L* = 5. The appropriate value of *k* is 3. Since 6 = (110)_2_, $Q_{3}$, $Q_{2}$ and ${\bar{Q}}_{1}$ are fed to the input pins of G3. When CNT1 reaches the state “110”, the output *Reach_L_*1 of G3 becomes ‘1’. The *Reach_L_*1 remains ‘0’ before CNT1 reaches “110”.

When *Reach_L_*1 turns to ‘1’, the clock *clk*_0_ of DFF1 that is driven by the system clock *CLK* ORed with *Reach_L_*1, is disabled. At this time, the output of the AND gate G4 driven by *TC* and the logical NOT of *Reach_L_*1, is ‘0’. Hence, the enable input *EN* fed by G4 is ‘0’ and the CNT1 is disabled. From the (*L +* 1)^st^ clock cycle, the CNT1 remains its state (i.e., *L +* 1), *Reach_L_*1 remains ‘1’ and the clock *clk*_0_ keeps inactive. The logical AND of the test key bits will be latched in DFF1 until the system is reset. If the correct test key is applied, the signal *Match* will remain ‘1’. From the (*L +* 1)^st^ clock cycle, the signal *unlock* driven by the logical AND of *Match* and *Reach_L_*1 will be ‘1’. If the incorrect test key is loaded, the signal *Match* will remain ‘0’ and the signal *unlock* is always ‘0’. 

As shown in Figure 9, the *unlock* signal controls the two output of the automatic test control unit via a NOT gate and an AND gate. When *unlock* = 1, both *Sig_Isol* and *Sig_Clear* will be zero regardless of the state of the automatic test control unit. When *unlock* = 0, the automatic test control unit will be enabled and can determine the values of both *Sig_Isol* and *Sig_Clear* as described in Section 2. 

Once a system reset is performed, the CUT, the CNT1 and the automatic test control unit are all reset. After a system reset, if the circuit first enter into the test mode (i.e., *TC* = 1), the test authorization key must be loaded before implementing the testing. If the circuit first enter into the normal mode (i.e., *TC* = 0) after a system reset, the CNT1 is disabled due to the zero value of *EN* and remains all-zero state. *Reach_L_*1 = 0 and *unlock* = 0, which enables the automatic test control unit. When the circuit is switched from the normal mode to test mode, the scan chain will be cleared. At the same time, the test authorization key should be loaded. The test key only needs (also is only allowed) to be applied once after a system reset. Once the correct test key is loaded at the beginning of testing, the standard test can be conducted on CUT in the following time. The scan chain will not be reset when switching mode and the cipher key will not be isolated in test mode. On the contrary, if the test key is incorrect, only the secure scan test can be performed.

## 5. Performance Analysis

The performance analysis of the proposed scheme consists of three aspects: testability, security and overhead.

### 5.1. Testability Analysis

In the proposed ISSATCU design scheme, the additional protection logic including the automatic test control unit and test authorization mechanism is independent of the original cryptographic module. Once the correct test authorization key is applied by the tester, the testing for the cryptographic module can be carried out normally. All types of test sets such as stuck-at fault test set and launch-on-shift/launch-on-capture based delay fault test set, can be used without any modifying. Furthermore, the detection efficiency of these test sets won’t be decreased. For the authorized users, the protection to the cipher key is removed and thus the online testing can also be performed. Therefore, the testability of the original crypto chip is not compromised by the proposed test scheme. However, faults in the introduced protection logic cannot be detected by the original test patterns. This issue can be resolved by using a built-in self-test (BIST) to test the small additional logic. As a widely utilized DFT methodology besides scan design, BIST can generate test patterns and analyze the test responses on chip. As a matter of fact, it is not essential to take into account the testing of the additional protection logic. If some defects exist in the additional protection logic, the test responses of some test patterns will mismatch with the expected values even though the original crypto module is fault-free and the right test key is applied. Under this situation, the CUT can be identified as faulty chip. In this sense, it won’t bring bad impact on the testability of CUT to ignore the testing of the additional protection logic.

### 5.2. Security Analysis

The security performance of the proposed ISSATCU scheme will be discussed under typical scan attacks:

(1) Brute force attack

For the attackers without any knowledge of the additional protection logic, the probability of hitting the *L*-bit test authorization key by chance is 1/2*^L^*. If a 128-bit authorization key is used, the probability of guessing the correct authorization key is as low as 2.94 × 10^−3^^9^. Even though *L* = 64, this probability is only 5.42 × 10^−20^. Hence, brute force attacks that attempt the test authorization key using the exhaustive search method is not feasible in theory. In specific application, the length of test authorization key ought to be set on the basis of acceptable area penalty and brute force probability.

(2) Mode switching attack

If the attackers fail to crack the test authorization key, they can only perform the secure scan test as described in Section 2. The circuit will be reset when switching from normal mode to test mode, so the attackers are not able to shift out and observe the intermediate encryption results generated in the normal mode. Consequently, the attacks based on mode switching can be overcome.

(3) Test-mode-only attack

When the circuit is running in test mode, the cipher key is masked from encryption module. In this way, the states of scan chains are not associated with the cipher key. The attackers can observe the content of scan chains by performing scan shift, but they cannot deduce the cipher key based on the observed data. Hence, the test-mode-only attacks cannot be carried out as well. The proposed technique eliminates any opportunity for scan-based non-intrusive attacks and has strong ability to protect the security of cryptographic chips.

### 5.3. Overhead Analysis

To evaluate the area penalty, the presented ISSATCU scheme is performed on pipelined and iterative AES designs with encryption key scheduling [38]. First, we synthesize the original AES cores by using Synopsys Design Compiler and gain their netlists. Then, the standard scan design is obtained by inserting scan chains into netlists with Synopsys Test Compiler. Finally, we insert the ISSATCU scheme into the scan design netlists and synthesize them by Synopsys Design Compiler. The results of the experiments are given in Table 1. The areas in the table are expressed with the number of equivalent 2-input NAND gates. The proposed ISSATCU scheme exploits five different lengths of test authorization key: 64, 80, 96, 112 and 128 bits. 

**Figure 10:**
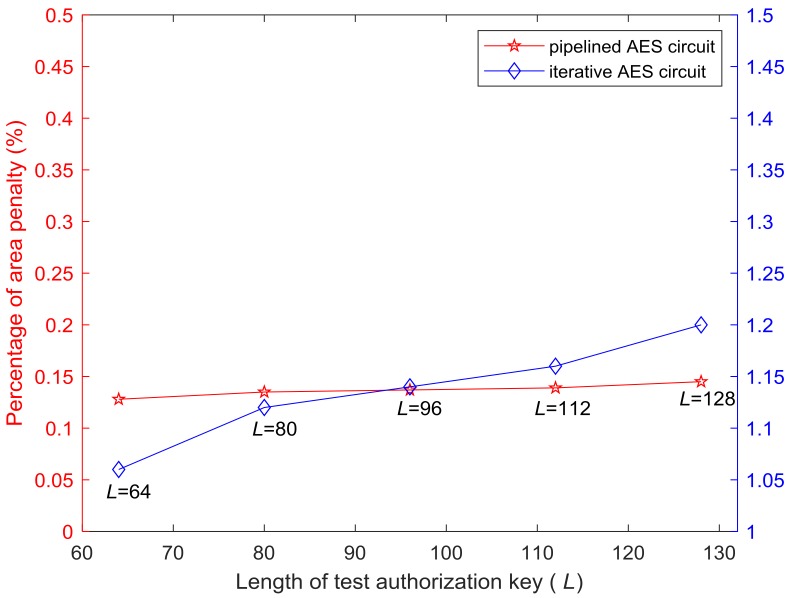
Relationship curve between the area penalty and the length of test authorization key.

For each length of test authorization key, the area overhead incurred by ISSATCU is described in Table 2. The last column presents the area penalty in percentage compared with standard scan design. In order to visually display the relation between the area penalty and the length of test authorization key (*L*), the percentage penalties in Table 2 are illustrated with Figure 10. As can be seen from Figure 10, for either pipelined or iterative AES circuit, the area penalty incurred by ISSATCU increases very slowly with the increase of *L*.

The presented technique is as well compared with other scheme protecting cryptographic chips against scan-based attacks, such as MKR [28], mode switching reset [27], secure DFT method [29], SOSD-128 [38], DOSD-128 [38], DOS [41], SIE [45], and FTSL-128 [40]. The comparison results are given in Table 3. MKR refers to the secure scan design based on mirror key register [28]. SOSD-128 and DOSD-128 represent the countermeasures based on the static and dynamic obfuscations of scan data in [38] with 128-bit obfuscation key, respectively. DOS refers to the dynamically obfuscated scan technique in which XOR gates are inserted behind some selected scan cells and the values of these scan cells are XORed with the state of an LFSR [41]. SIE represents scan interface encryption technique [45]. FTSL-128 represents the field test scan lock solution using PUF in [40] with 128-bit test key. 

Compared with other protection strategies, the area overhead of ISSATCU is relatively low and completely acceptable. The proposed ISSATCU scheme provides high security for crypto chips because it can thwart all known scan-based non-intrusive attacks. It does not hurt the testability of chips as well. The only drawback is that the extra test time is required to input test authorization key before testing. If the scan cells storing the authorization key lie in a same scan chain, the test time overhead is 128 clock cycles for ISSATCU with 128-bit authorization key. If the authorization key are distributed into multiple scan chains, the test time overhead will be less than 128 clock cycles. The secure DFT [29] and MKR [28] incur very low area overhead and no test time overhead, and make brute force attack useless. Nevertheless, they restrict the online testing. Since the lines loading the encryption key from non-volatile memory to round key register cannot be tested, the testability of chips is hurt too. The mode switching reset technique [27] possesses the similar advantages and disadvantages with secure DFT [29] and MKR [28] except for large area overhead. The DOSD countermeasure [38] has high security with the test time overhead of 128 clock cycles. The shortcoming is that, LOC delay testing cannot be implemented on circuits protected by DOSD. SOSD countermeasure [38] incurs less area overhead than DOSD countermeasure, but has relatively weak security as it’s not resistant to test-mode-only attack. The DOS countermeasure [41] results in large area penalty and it’s not resistant to the memory attack. The SIE methodology [45] maintains the testability of CUT with large hardware overhead. The brute force probability of the SIE depends upon the key length of block cipher. The SIE is vulnerable to the memory attack and needs multiple clock cycles to decrypt test data during testing. The security of FTSL design is high, but it incurs very large area penalty [40]. Furthermore, it limits the application of the LOC delay testing. We can see from Table 3 that the proposed ISSATCU surpasses the existing schemes in one or more attributes. In general, the ISSATCU can provide effective protection for crypto chips with low area and test time overhead without compromising the testability of chips.

## 6. Conclusions

Adversaries can employ the side channel offered by scan design to deduce the secret key based on intermediate results of the encryption operation. In this paper, we present a secure strategy based on test authorization and intelligent test control. This strategy gives the authorized users the privilege to perform the normal test. For an unauthorized user, the intelligent test control unit is activated to manage the behaviors of the crypto chip. When the chip runs in normal mode, the encryption operation can be implemented normally. Once the chip is switched from normal mode to test mode, the intelligent test control unit will reset the system to clear the sensitive data stored in scan chains. When the chip enters the test mode, the intelligent test control unit isolates the cipher key from crypto module to prevent the leakage of the secret information. The proposed countermeasure is resistant to existing noninvasive scan attacks while it does not decrease testability of original scan design. All types of tests including the test of stuck-at fault and transition-delay fault are still applicable. Experimental results also show a small overhead of area and test time. The presented countermeasure outperforms all existing secure designs in most of characteristics. It is especially applicable to protect cryptographic chips in resource-constrained environments such as WSN.

## Figures and Tables

**Table 1:** Areas of original circuit, standard scan design and proposed ISSATCU.

AES Circuit	Area: The Number of Equivalent 2-Input NAND Gates						
	Original Circuit	Standard Scan Design	ISSATCU				
			*L* = 64	*L* = 80	*L* = 96	*L* = 112	*L* = 128
pipelined	205,934	212,280	212,551	212,567	212,571	212,575	212,587
iterative	25,052	25,512	25,783	25,799	25,803	25,807	25,819

**Table 2:** Percentage area penalty of proposed ISSATCU.

AES Circuit	ISSATCU	Area Penalty	ΔArea Percentage
pipelined	*L* = 64	271	0.128%
	*L* = 80	287	0.135%
	*L* = 96	291	0.137%
	*L* = 112	295	0.139%
	*L* = 128	307	0.145%
iterative	*L* = 64	271	1.06%
	*L* = 80	287	1.12%
	*L* = 96	291	1.14%
	*L* = 112	295	1.16%
	*L* = 128	307	1.20%

**Table 3:** Comparison of different security schemes.

Secure Schemes	Area Penalty (%)		Security		Impact on Test Time	Limit on Test Application
	Pipelined	Iterative	Vulnerability	Brute Force Probability		
ISSATCU with 128-bit authori-zation key	0.15	1.20	None	2^−128^	less than or equal to 128 clock cycles	All types of tests are applicable
Secure DFT [29]	0.11	0.96	None	inapplicable	No extra clock cycles	Online testing is inapplicable
MKR [28]	0.15	1.32	None	inapplicable	No extra clock cycles	Online testing is inapplicable
Mode switching reset [27]	≈10	--	Test-mode- only attacks	inapplicable	No extra clock cycles	Online testing is inapplicable
SOSD-128 [38]	0.34	2.81	Test-mode- only attacks	2^−128^	128 clock cycles before testing	LOC Delay testing is inapplicable
DOSD-128 [38]	0.47	3.91	None	2^−128^	128 clock cycles before testing	LOC Delay testing is inapplicable
DOS [41]	2.01	--	Memory attack	2^−*kλ*^ *	No extra clock cycles	All types of tests are applicable
SIE [45]	2.52	--	Memory attack	2^−*m*^ **	multiple clock cycles for vector decryption	All types of tests are applicable
FTSL-128 [40]	3.80	31.66	None	2^−128^	128 clock cycles before testing	LOC Delay testing is not applicable

* *k* and *λ* denote the number and the length of scan chains. ** *m* denotes the key length of block cipher.
